# Suboptimal control status of young hypertensive population

**DOI:** 10.1186/s40885-023-00237-6

**Published:** 2023-05-01

**Authors:** Donghun Shin, JungMin Choi, Hae-Young Lee

**Affiliations:** 1 Seoul National University College of Medicine, Seoul, Republic of Korea; 2 Division of Cardiology, Department of Internal Medicine, Seoul National University Hospital, Seoul, Republic of Korea; 3 Department of Internal Medicine, Seoul National University College of Medicine, Seoul, Republic of Korea

**Keywords:** Hypertension, Young adults, Blood pressure

## Abstract

The prevalence of hypertension (HT) among young adults aged 18 to 39 years is estimated to be 3.7% to 8.6% worldwide. Although the prevalence of HT in young adults is lower than that of the overall population, those with HT are at substantially increased risk of cardiovascular events compared to those without HT. HT in young adults should be taken with even more caution as longer exposure to higher blood pressure leads to a higher lifetime risk of HT-mediated organ damage. However, young patients with HT show low awareness of HT compared to older patients. Also, they are more prone to show low treatment adherence despite the good efficacy of the treatment. Other risk factors that hinder HT control among young adults include alcohol intake, smoking, low physical activity, emotional stress, job stress, metabolic syndrome, and obesity. This review aimed to illustrate the suboptimal control status of the young hypertensive population and to propose strategies for improvement.

**Supplementary Information:**

The online version contains supplementary material available at 10.1186/s40885-023-00237-6.

## Background

Hypertension (HT) is considered a disease of older adults, due to which, cardiovascular risk assessment and active control have been suboptimal for younger hypertensive populations [1]. In South Korea, approximately 90% of the young hypertensive population is unaware that they have HT [2] and only 24% of them are treated [3]. Although national guidelines have been implemented adequately in older hypertensive patients, several recent meta-analyses and reviews have reported a low degree of compliance with these guidelines in young hypertensive patients [4–10]. Obstacles in HT control includes physician inertia, defined as the inability to control treatment despite failure to achieve the blood pressure (BP) target, patient's reluctance, and low compliance [11–13]. Here, we discuss the epidemiology of HT, socio-medical risk factors which might hinders HT control, and cardiovascular event rates in young hypertensive patients and propose strategies to enhance the management of HT in young adults ≥ 20 years old. HT was defined as an SBP/DBP ≥ 140/90 mmHg without antihypertensive treatment or taking antihypertensive treatment.

## Epidemiology of hypertension among young adults

Among the general population, the age-standardized prevalence of HT in 2019 was reported to be 34% in men and 32% in women [14]. In Korea, data from the Korea National Health and Nutrition Examination Survey (KNHANES) reported that the age-standardized prevalence of HT among adults aged ≥ 20 years decreased from 26.0% in 1998 to 22.2% in 2019 [3]. However, with the rapid aging of the population, the absolute number of people with HT has steadily increased with more than 12 million at 2019.

The prevalence of HT among the younger generation is much lower than that in the overall population. The prevalence among young adults was estimated to be 9.5%, resulting in 1.27 million young adults being affected. The prevalence of HT in young adults differ depending on the sex. Although such disparity among sex is a common phenomenon, the pattern of difference differs compared to the older population. In middle aged population, the prevalence of HT is similar between different sexes and the HT prevalence rises higher in women compared to men when they get older. In younger generations under 40 years of age, the prevalence of HT in men is approximately 15%, but that in women is less than 5% [3]. Similarly, in the United States, according to the National Health and Nutrition Examination Survey (NHANES) 2015–2016 data, the prevalence among young adults aged 18 to 39 years was 7.5%, which was subdivided into 9.2% in men and 5.6% in women [14]. In Japan, an analysis of data from the National Health and Nutrition Survey reported that the prevalence of HT ranged from 10.2% to 18.1% in young men and 4.1% to 4.5% in young women in 2016 [15].

Another remarkable finding is the high prevalence of prehypertension among young adults. According to the Korean Hypertension Face Sheet 2020, nearly one-third of young adults over 20 years of age (3,387,000 in 10,536,000) have prehypertension with systolic BP (SBP) 130 to 139 mmHg and diastolic BP (DBP) 80 to 89 mmHg [16]. If they are not adequately controlled, a considerable portion of the prehypertensive population will progress to the hypertensive range with aging.

The most important problem in the younger hypertensive population is that more than 90% of them are not aware that they have HT. The awareness rates of HT have improved little in younger adults from 10% in 1998 to 15% in 2019, which is in sharp contrast to older adults, whose awareness rates have steadily increased from 30 to 80% in the same period in Korea [3]. As a result, only 24% of young hypertensive patients seek medical care at least once and among them, only one-third are regularly treated [2]. Similarly, the US NHANES 2013–2014 data reported an awareness rate of 74.7%, a treatment rate of 50%, and a control rate of 40.2% among young adults with HT, which was lower than that of hypertensive patients in the all-age group (84.4%, 74.7%, and 53. 9%, respectively) [17].

Conversely, young hypertensive patients showed better control rate than older adults when treated. Here, control rate is defined as the proportion of patients who achieved the target BP among those who are receiving treatment, as denoted by control^2^ in Table 1.

**Table 1:** Prevalence, awareness, treatment, and control of hypertension according to country, age, and sex

Country	Age^a^ (yr)	Sex	Prevalence^b^ (%)	Awareness (%)	Treatment (%)	Control^1^ (%)	Control^2^ (%)
USA	All	Both	29.0 [18]	84.4 [17]	74.7 [17]	53.9 [17]	73.0 [17]
		Male	30.2 [18]	NS	NS	NS	NS
		Female	27.7 [18]	NS	NS	NS	NS
	18–39	Both	7.5 [18]	74.7 [17]	50.0 [17]	40.2 [17]	80.4 [17]
		Male	9.2 [18]	NS	NS	NS	NS
		Female	5.6 [18]	NS	NS	NS	NS
Korea [3]	All	Both	22.2	67.3	63.6	46.2	72.0
		Male	25.5	NS	NS	NS	NS
		Female	18.5	NS	NS	NS	NS
	19–29	Both	5.2	NA	NA	NA	NA
		Male	6.4	NA	NA	NA	NA
		Female	3.9	NA	NA	NA	NA
	30–39	Both	9.5	20.2	15.2	9.8	64.7
		Male	15.1	NS	NS	NS	NS
		Female	3.4	NS	NS	NS	NS
	20–39	Male	NA	23.4	11.7	8.0	68.2
		Female	NA	20.7	20.7	17.6	84.9
Japan	All	Both	48.9 [19]	67.0 [15]	56.0 [15]	27.0 [15]	48.2 [15]
		Male	40.3 [14]	65.8 [14]	45.9 [14]	24.1 [14]	52.5 [14]
		Female	22.5 [14]	68.8 [14]	51.2 [14]	30.3 [14]	59.2 [14]
	20–34	Male	16.2 [20]	NA	5.3 [20]	3.4 [20]	65.0 [20]
		Female	4.1 [20]	NA	17.6 [20]	15.2 [20]	86.4 [20]
	35–49	Male	35.5 [20]	NA	12.9 [20]	3.3 [20]	25.8 [20]
		Female	20.4 [20]	NA	17.6 [20]	5.9 [20]	33.3 [20]
China [21]	All	Both	23.2	46.9	40.7	15.3	37.5
	18–24	Both	4.0	5.7	3.4	0.6	17.9
	25–34	Both	6.1	14.7	8.4	3.2	37.1
	35–44	Both	15.0	31.7	24.5	9.9	40.3
Greece [22]	All	Both	39.6	68.2	65.6	30.5	46.5
	18–29	Both	7.1	11.1	11.1	6.9	62.2
	30–39	Both	13.1	14.9	9.4	5.1	54.3
Ghana [23]	All	Both	13.0	45.6	40.5	23.8	58.8
	15–24	Both	3.7	38.0	80.9	75.0	92.7
	25–34	Both	11.1	45.1	85.9	61.8	71.9
	35–44	Both	21.8	46.2	90.1	47.4	52.6
	45–49	Both	13.0	45.6	40.5	23.8	58.8

Hypertension was defined as systolic blood pressure of at least 140 mmHg and/or diastolic blood pressure of at least 90 mmHg and/or the use of antihypertensive medication. Control^1^ is the rate among individuals with hypertension. Control^2^ is the rate among individuals under hypertension treatment. Because the accessibility of data varies from country to country, if the data is not accessible, the corresponding field is left blank. If one of the two control rates was not accessible, it was calculated using the following equation:

$$\frac{\mathrm{Control rate among individuals with hypertension }(\mathrm{\%})}{\mathrm{Treatment rate }(\mathrm{\%})} =\mathrm{ Control rate among individuals under hypertension treatment }(\mathrm{\%})$$

*NS* not specified, *NA* not available, *KOSIS* Korean Statistical Information Service, NCD-RisC NCD Risk Factor Collaboration

^a^The all-age groups represent the general population in each country and were defined as follows: ≥ 18 in the United States, Korea, China, and Greece,  ≥ 20 in Japan, and 15–49 in Ghana; ^b^For all-age groups, prevalence was age-standardized except for Satoh et al. [19]

In the Korean Hypertension Face Sheet 2021, young hypertensive patients had lower control rate than all-age hypertensive patients in overall (9.8% vs. 46.2%). However, among those treated, young hypertensive patients showed control rates similar to that of older individuals (68.2% in males aged 20–39 years vs. 69.8% in males aged 50–59 years; 84.9% in females aged 20–39 years vs. 79.7% in females aged 50–59 years) [3]. The US NHANES 2013–2014 data also reported a higher control rate among young hypertensive patients than in the overall population (80.4% vs. 73.0%) [17]. Similarly, prevalence of masked HT is reported to be about 15% in the general population, but was higher in young adults, suggesting low treatment adherence [4, 24]. Table 1 summarizes the prevalence, awareness, treatment, and control rates in young adults and the overall population in various countries.

## Socio-medical risk factors hindering hypertension control among young population

**Fig. 1:**
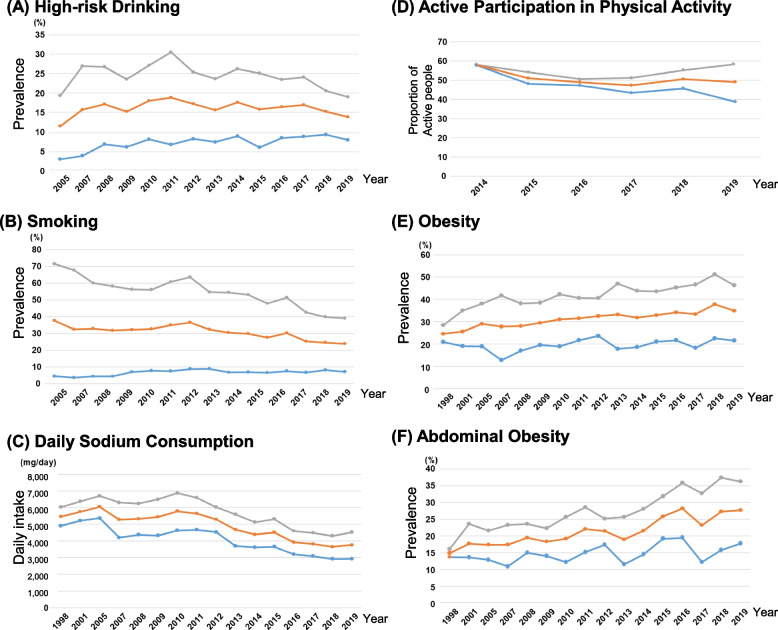
Trends in socio-medical risk factors hindering hypertension among Korean young adults aged 30–39 years produced based on Korea National Health and Nutrition Examination Survey 1998–2019. **A** Proportion of high-risk drinking, **B** smoking rate, **C** daily sodium consumption, **D** active participation in physical activity, **E** obesity rate, and **F** abdominal obesity rate

Various socio-medical risk factors not only lead to HT development but also hinder HT control in young adults. The trends in socio-medical risk factors among Korean young adults aged 30 to 39 years based on KNHANES 1998–2019 are described in Fig. 1. The risk factors included are alcohol consumption, smoking, sodium intake, physical activity, emotional stress, metabolic syndrome, and obesity. The specific definition of each risk factors is described in Supplementary Table S1.

Regarding alcohol consumption, men were more likely to be high-risk drinkers than women (20% vs. 10%). In the age group of 30 to 39 years, men were more likely to be smokers than women (40% vs. 10%), despite the recent substantial decrease of smoking population in men. Although sodium intake has also decreased continuously, daily sodium intake in young adults is still high as 10 g/day and is higher than that in older adults.

Albeit decrease in sodium intake, alcohol consumption, and smoking, physical exercise and obesity have also decreased as well among young adults. The physical activity of young men increased from 2016 to 2019, whereas that of young women decreased in the same period. A study using self-reported questionnaires and accelerometers found that young adults spend more time of their day in sedentary work, than the whole population. There was a significant association between physical fitness and HT in a longitudinal cohort study of young adults aged between 18 and 30 years [25]. In the study period of 15 years, those in the bottom two deciles of fitness, as measured by the duration on a treadmill test, were twice as likely to have HT as those in the top four deciles. In addition, in the 7-year follow-up period, improving physical fitness compared to the baseline reduced the risk.

Another problem is the increased prevalence of obesity and metabolic syndromes among the young adults. In a meta-analysis of four prospective cohort studies, individuals who were consistently obese from a young age have a 2.7 times higher risk of developing HT as compared to consistently non-obese patients [26]. The current prevalence of metabolic syndrome among Korean adults aged between 19 to 29 years is 5.9% and 27.7% among adults aged over 30 years. Recently, obesity in children (mean age, 13.7 years old) has risen from 6 to 10%during 2008 to 2019. Such increase in child obesity is likely to result in obese young adults as those who are obese during childhood are likely to remain obese when they become adults [27]. The prevalence of obesity in young adults aged 19–29 was 15.2% and 27.6% in 1998 and in 2019, respectively. Similarly, the prevalence of metabolic syndrome in young adults is also increasing. The incidence of abdominal obesity among young adults has increased significantly from 15% in 1998 to 27% in 2019 [28]. By such increase in obesity is likely to result in more hypertensive young adults.

## Hypertension-mediated cardiovascular events among young population

HT is an important cardiovascular risk factor even in young adults. Recently, the association between office BP levels and major cardiovascular events in 98,000 young hypertensive patients (< 50 years old) was evaluated, using the Korean National Health Insurance Service database [29]. During a mean follow-up of 9.5 ± 2.8 years, 4,918 (5%) major adverse cardiac events (MACEs) were documented in the young HT cohort. Elevated BP levels (< 120/ < 70 mmHg) were significantly correlated with an increased risk of MACE in younger Korean hypertensive patients. Similarly, Son et al. [30] reported that young men aged 20 to 39 years old with prehypertensive BP range (130–139/80–89 mmHg) had a higher risk of cardiovascular disease when compared with those having normal BP (< 120/ < 80 mmHg; incidence, 215 vs. 164 per 100,000 person-years; adjusted hazard ratio, 1.25; 95% confidence interval [CI], 1.21–1.28), coronary heart disease (incidence, 134 vs. 103 per 100,000 person-years; adjusted hazard ratio, 1.23; 95% CI, 1.19–1.27), and stroke (incidence, 90 vs. 67 per 100,000 person-years; adjusted hazard ratio, 1.30; 95% CI, 1.25–1.36). Women with the same BP range also had an increased risk of cardiovascular disease (incidence, 131 vs. 91 per 100,000 person-years; adjusted hazard ratio, 1.27; 95% CI, 1.21–1.34), coronary heart disease (incidence, 56 vs. 42 per 100,000 person-years; adjusted hazard ratio, 1.16; 95% CI, 1.08–1.25), and stroke (incidence, 79 vs. 51 per 100,000 person-years; adjusted hazard ratio, 1.37; 95% CI, 1.29–1.46).

Moreover, a longer HT morbidity duration leads to a higher lifetime risk of HT-mediated organ damage. The onset of HT before the age of 35 years was significantly associated with increased risk of left ventricular hypertrophy, coronary calcification and diastolic dysfunction compared to the onset of HT over the age of 45 years, in the follow-up period of 25 years [31]. Also, in a prospective study of 3,381 adults (age, 18–30 years at baseline) with 25 years of follow-up, higher cumulative SBP and DBP and fasting blood glucose were consistently associated with cognitive impairment in middle age [32].

BP patterns tend to change with age. As DBP tends to decrease with age, it is usually considered as an important risk factor of cardiovascular events in younger adults, whereas systolic BP is considered more important in older age [33]. However, isolated systolic HT (defined as SBP > 140 mmHg and DBP < 90 mmHg) which is especially common in young males [34], was associated with future development of sustained HT as well [35]. With such perplexing results, guidelines have not yet reached consensus on the treatment policy for isolated systolic HT in young patients [36]. However, a scoping review in 2021, encompassing 20 studies, identified important predictors of cardiovascular risk of isolated systolic HT in young males, suggesting drug treatment in high-risk young adult patients [34].

## Strategies to enhance management of young hypertensive population

**Fig. 2:**
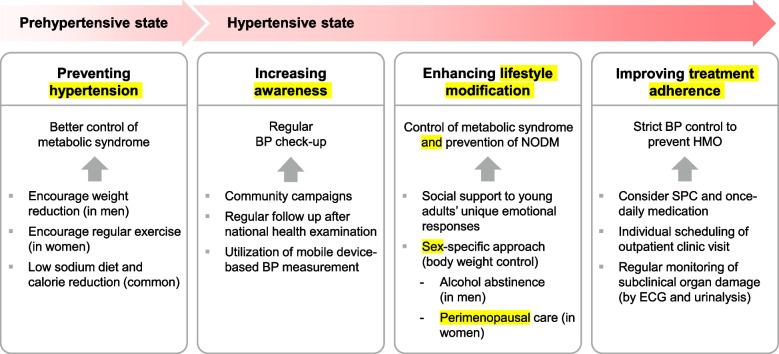
Strategies to enhance current management status of young population with high blood pressure (BP). NODM, new onset diabetes mellitus; HMO, hypertension-mediated organ damage; SPC, single pill combination; ECG, electrocardiogram

In the last section, we proposed a strategy to optimize HT control among adults in four aspects: HT prevention, increasing awareness, enhancing lifestyle modification, and increasing treatment adherence. The proposed method is illustrated in Fig. 2.

The first is the primary prevention of HT. For prevention of HT development, control of metabolic syndromes is essential. Although there is no debate that lifestyle modification is important, current guidance on lifestyle modification is unlikely to maintain long-term effects [37]. There is a need for continued research to improve age- and sex-specific strategies and recommendations for young adults. In both men and women, reduced sodium intake should be advocated, considering that sodium intake in young adults is higher than that in older adults. In case of young women, body weight continuously decreases, but the physical activity of women also decreases in the same period. Lack of exercise increases the risk of metabolic syndrome in young women [38].Vigorous exercise for 20 min. or more per week is recommended to prevent HT in young women in a prehypertensive range. Obesity is the most common phenotypic characteristic of young adults with HT. Returning to normal weight reduces the risk of HT to a level comparable to that in people with normal weight [26]. Thus, the importance of vigorous exercise and weight loss in obese patients to normal weight should be more emphasized. Another consideration needed regarding obesity, it that the obesity prevalence is high in lower income and education groups, suggesting that socioeconomic disparities in obesity have widened [39]. Therefore, social support to ensure mealtimes and a balanced diet for low-income families should be promoted.

The second point is the increasing awareness. Increasing awareness is particularly important for young adults, whose awareness rates are lower than those of older individuals. The first step is to encourage young adults to regularly measure their BP. Several initiatives have been undertaken to raise awareness in the general population. For example, the May Measurement Month (MMM) campaign, an annual initiative held by the International Society of HT, aims to raise awareness of HT by inspiring adults to have their BP measured at community centers and to complete a survey on risk factors for high BP [40]. Furthermore, on-line campaign such one-line MMM program or information using YouTube or social media by academic society or by governmental institutions may be more important in the young population to increase the exposure to information regarding HT. In addition, regular follow-up for elevated BP after a national health examination is important for young adults. Another promising strategy is to utilize mobile devices that are being widely used by young adults. Researchers have actively explored the potential role of mobile device-based BP measurements in enhancing the awareness, treatment, and control of HT. In 2020, the Korean Society of Hypertension published its position papers specifically for cuffless BP-measuring devices in 2021, and the European Society of Hypertension published a consensus document in 2022 [41, 42]. The authors stated an ancillary benefit of the calibration process, which mandates the user to regularly measure BP using conventionally accepted methods. This is supported by the fact that most cuffless BP- measuring technologies are based on pulse transit time or pulse wave analysis, which requires the user to calibrate cuff BP measurements at regular intervals [43]. However, there are many issues to be solved when using a cuffless BP monitoring device in hypertensive patients, mainly regarding the optimal validation protocol. Notwithstanding, academic societies pay attention to the high potential of cuffless BP-measuring devices for raising awareness and early detection of HT, especially in young adults whose awareness of their BP is lower than that of the older population.

The third point is the enhancement of lifestyle modifications. It is needless to say that lifestyle modification is the most important strategy to resolve metabolic syndrome. Core principles of lifestyle modification are stated consistently in previous guidelines, which include dietary modification, weight reduction, regular physical activity, and reduction of alcohol and sodium consumption [4, 10]. However, it is also a stark fact that non-individualized, one-time education has shown limited long-term effects on healthy lifestyle maintenance. Therefore, it is important to evaluate sex and age group-specific socio-medical risk factors, including alcohol abstinence in men and peri-menopausal care in women. Kim et al. [44] analyzed sex-specific risk factors in 780 young hypertensive patients. For men, factors included hyperlipidemia, marital status, and family history of HT; for women, factors included diabetes mellitus, job stress, and high sodium intake. It is also important to understand young adults’ unique responses to HT, not only as a clinical aspect but also as a social aspect of the young patients. A multi-center qualitative study was proposed to aid clinicians in understanding young adults' perspectives upon the diagnosis of HT [45]. HT diagnosis, education regarding lifestyle modification, and drug therapy damages the “young” identity of the patients [45]. Understanding and protecting such identity of the patient is an important factor in the management of chronic illness [46]. Johnson et al. [45] emphasized the development of interventions that specifically target young adults’ unique emotional responses.

Another problem in patient education of young hypertensive patients is that cardiovascular risk is often low due to young age. Because cardiovascular risk stratification according to current guidelines is based on 10-year cardiovascular model, a young adult without clear evidence of organ damage is often assessed as a low-risk patient. However, their lifetime risks substantially increase, which is not reflected in the current cardiovascular risk stratification system [9]. Young patients with many cardiovascular risk factors have a low absolute risk but a high relative risk, which is neglected in the current system [4]. This can be misleading not only to clinicians, but also to patients, in that patients may underestimate their risks and belittle the importance of lifestyle modification and drug treatment. In this regard, the European Society of Hypertension guidelines proposed the use of a new way to communicate cardiovascular risk for young patients, which is called the “cardiovascular risk age.” In this system, a young patient with risk factors is represented as being of the age of an older patient without risk factors but with the same cardiovascular risk, which can be estimated automatically using HeartScore [4]. In addition, in communicating risk of young patients, it should be taken into consideration that the current systems are validated on data whose age range does not often fully include young adults, which makes its applicability questionable in case of young adults [47]. For example, the European guideline uses a system validated in the age over 40 years [4], British in the age range of 35 to 74 years [9], and Korean in the age range of 35 to 59 years [10].

The fourth point is improving adherence to treatment, which is directed toward strict control of BP to prevent HT-mediated organ damage. Adherence to pharmacological therapy is a crucial component of HT control [48]. However, adherence to antihypertensive medication is low among young hypertensive patients; about 58% being non-adherent, whereas older age groups demonstrated non-adherence rates ranging from lowest 24% to highest 47% in the United States [49]. Moreover, a recent study has demonstrated a dose–response relationship between lower adherence and higher cardiovascular disease risk; the non-adherent group had a 1.57 times higher cardiovascular disease risk than the adherent group [50]. To improve adherence, individual appointments for outpatient clinic visits are important. It has also been demonstrated that a single-pill combination (SPC) and once-daily medication are beneficial for drug therapy. A meta-analysis demonstrated increased adherence and persistence in patients of all ages treated with SPC as compared to those treated with a free-dose combination [51]. Another review on the effectiveness of once-daily medication also reported better adherence in the once-daily group than in the multiple-daily group [52]. Therefore, this can be especially beneficial for young hypertensive patients whose adherence has been demonstrated to be lower than that of older adults.

Regular monitoring of subclinical organ damage may be an effective way to increase adherence. Left ventricular hypertrophy can be easily assessed using electrocardiogram (ECG) at a low cost. Yet, clinician should be cautious when interpreting high ECG potentials as left ventricular hypertrophy as it can be also seen in young patients with structural heart problem [53]. In such cases, an echocardiographic study can help confirm left ventricular hypertrophy [54]. Another cost-effective evaluation tool is urinalysis, which can be used to assess albuminuria.

The last point is the importance of strict BP control of younger patients with HT. Some clinicians may question the need to strictly control BP using drug therapy in young patients with HT. However, it is important in young patients for the following reasons. First, as described in the previous section, a higher BP is associated with a substantially increased lifetime risk of HT-mediated organ damage. Second, the proportion of risk reduction associated with BP reduction was higher in younger adults, although the absolute risk was lower. This is supported by multiple meta-analyses on the relationship between cardiovascular disease risk and BP by age group [55, 56]. In the logarithmically linear relationship between the cardiovascular mortality risk and BP, the slope was higher in the younger age group. Third, the effect of drug use on increasing event-free life expectancy is higher in younger patients, whereas the effect of drug use on reducing short-term cardiovascular disease risk is higher in older patients, as demonstrated by an analysis of data from Individual Data Analysis of Antihypertensive drug intervention trials (INDANA) and national statistics [57]. Although there is limited evidence to confirm the benefit in randomized clinical trials, multiple observational studies have suggested that the benefit of intensive BP-lowering might be consistent among young hypertensive populations.

## Conclusions

The young hypertensive population is characterized by low awareness of HT but already has a high prevalence of prehypertension. Although the control rate among all hypertensive populations was low, that among the treated population was comparable to and even higher than that of the older population. Although cardiovascular disease risk calculated by current guidelines remain to be low due to young age, HT is associated with an increased risk of lifetime cardiovascular disease risk in young adults. To optimize HT control among young adults, we propose the following recommendations: prevention of HT by better control of metabolic syndrome, especially through weight reduction in young men and physical activity in young women; increasing awareness by regular BP check-ups; enhancing lifestyle modification aimed at prevention of new onset diabetes mellitus; and improving treatment adherence by strict BP control to prevent HT-mediated organ damage.

## Supplementary Information

**Additional file 1: Supplementary Table S1.** Definition of socio-medical risk factors.

## Data Availability

Not applicable.

## Abbreviations

BP: Blood pressure
CI: Confidence interval
DBP: Diastolic blood pressure
ECG: Electrocardiogram
HT: Hypertension
HMO: Hypertension-mediated organ damage
INDANA: Individual Data ANalysis of Antihypertensive drug intervention trials
KNHANES: Korea National Health and Nutrition Examination Survey
KOSIS: Korean Statistical Information Service
MACE: Major adverse cardiac event
NA: Not available
NCD-RisC: NCD Risk Factor Collaboration
NHANES: National Health and Nutrition Examination Survey
NODM: New onset diabetes mellitus
NS: Not specified
SBP: Systolic blood pressure
SPC: Single-pill combination
