# Supplementary material for: Suboptimal control status of young hypertensive population
Source: Clin Hypertens. 2023 May 1;29:13. doi: 10.1186/s40885-023-00237-6 (PMC10150511; doi:10.1186/s40885-023-00237-6)
Supplement: Supplementary file 1 — Additional file 1: Supplementary Table S1. Definition of socio-medical risk factors. [file 40885_2023_237_MOESM1_ESM.docx]

## Supplementary Table S1. Definition of socio-medical risk factors

| **Socio-medical risk factors** | **Definition** |
| --- | --- |
| **High-risk drinking** | >7 units per drink and >2 times per week for men  >5 units per drink and >2 times per week for women. |
| **Active participation in physical activity** | At least 2 hours and 30 minutes of moderate-intensity physical activity per week; or at least 1 hour and 15 minutes of high-intensity physical activity per week; or combined moderate- and high- intensity physical activity for time equivalent of each activity, by converting 1 minute of high-  intensity physical activity into 2 minutes of moderate-intensity physical activity. |
| **Obesity** | Body mass index over 25 kg/m^2^ |
| **Abdominal obesity** | Abdominal circumference of ≥90 cm for men, and abdominal circumference of ≥85cm for women. |
